# Implications of the latest release of the National Statement on Ethical Conduct in Human Research on health promotion practice in Australia

**DOI:** 10.1002/hpja.888

**Published:** 2024-06-06

**Authors:** Krysten Blackford, Gemma Crawford, Sharyn Burns

**Affiliations:** ^1^ Collaboration for Evidence, Research and Impact in Public Health (CERIPH), Curtin University Perth Western Australia Australia; ^2^ School of Population Health, Curtin University Perth Western Australia Australia

**Keywords:** capacity building, competencies, ethics, evidence based practice, workforce development

The National Health and Medical Research Council (NHMRC) promotes ethical conduct and research integrity by providing practical guidelines and advice for Australian researchers.^1^ The NHMRC released the National Statement on Ethical Conduct in Human Research 2023 (National Statement) in July 2023.^2^ The 2023 National Statement takes effect from 1 January 2024, replacing the 2007 National Statement.^3^ This set of guidelines is intended for use by anyone conducting research with human participants; members of ethics committees; anyone involved in research governance; and potential research participants.^1^ We explore the implications of these updated guidelines for health promotion practice in Australia through our collective experiences as members of the Australian Health Promotion Association (AHPA) Working Group for the Health Promotion Ethics Project (HPEP), Associate Editor of the Health Promotion Journal of Australia (HPJA), Member National Accreditation Organisation Management Committee (AHPA) and Chair of a University Human Research Ethics Committee.

For many years, the HPJA has had a strong focus on ethical health promotion practice, including a dedicated special issue. In their editorial, Carter et al.^4^ reflected on the issue of HREC approval for health promotion research, evaluation and quality assurance. The editors argued for the National Statement's importance, concluding that concerning health promotion research broadly and the work of the HPJA more specifically, the National Statement should guide practice at all stages of health promotion activity. At this time, the HPJA introduced more robust guidelines for the application and reporting of ethical oversight to HPJA papers reporting on ‘research‐like activities’ with exceptions made rarely.^4^

Although not always framed as research, health promotion work is usually conducted with communities and often involves collecting data from ‘human participants’ for evaluation or quality assurance purposes. Therefore, as our previous formative research with health promotion practitioners and organisations has established, ethical health promotion practice should be underpinned by practitioners' understanding of ethical guidelines, application of critical health promotion values and principles, and, where appropriate, access to ethical oversight via a human research ethics committee (HREC) or via a recognised quality assurance review.^5^, ^6^ To support ethical practice, the literature suggests that health promotion practitioners require an understanding and awareness of ethical frameworks and considerations for practice to assist with evidence‐informed decision‐making and priority‐setting.^7^, ^8^ Despite this, we have found practitioners and organisations often face various individual, organisational, and structural barriers to ethical practice.^5^, ^9^ These include a lack of awareness and use of ethical frameworks and limited guidelines to support meaningful engagement with priority populations.

As Australia's peak health promotion body, AHPA aims to support ethical health promotion practice via the HPEP^10^ (see https://www.healthpromotion.org.au/ethical-health-promotion). In response to the barriers to ethical practice described above,^5^ the HPEP aims to provide opportunities to build AHPA's capacity to lead conversations about ethical health promotion practice with members, build sector knowledge and skills and facilitate access to formal Human Research Ethics support for health promotion evaluation and research.^10^ Integral to the project is facilitating an improved understanding of guidelines that provide a foundation for ethical conduct and integrity, such as the 2023 National Statement,^2^ and promoting the ethical values outlined in the Core Competencies and Professional Standards for Health Promotion.^11^

The revised version of the National Statement includes updates to chapter 2.1, which focuses on Risk and Benefit. The major change is moving to a continuum‐based model from high to minimal risk, divided into the broad categories of higher and lower risk.^2^ The previous model incorporated three levels of risk (greater than low risk, low risk and negligible risk) determined by subjective and imprecise distinctions between harm, discomfort and inconvenience that were open to interpretation by researchers and reviewers.^12^ The revisions distinguish between discomfort and harm and recognise that harm can be experienced individually or collectively. Inconvenience and burden to participants are also included on this continuum. The new guidelines support researchers, institutions, and reviewers to determine the risk level according to internal policies and practices. A barrier identified in HPEP's formative research was participants' perception that HREC assessors lacked understanding of the scope and context of health promotion practice.^5^ While assessors do not need to be experts in every discipline, the research suggested that limited knowledge of community‐level health promotion work, population health approaches more broadly, and diverse cultures and population groups presented challenges during the review process. For example, participants cited ‘grassroots community development participatory approaches that do not fit the model of a traditional ethics approval process’.^5^ The update to chapter 2.1 of the National Statement will facilitate nuanced risk assessment that can support ethical approval processes for health promotion practice.

There are also updates to section 5, which focuses on Research Governance and Ethics Review. Changes to this section include amendments associated with risk levels to align with the updates to chapter 2.1, including updated review pathways and eligibility for exemption from ethics review; and new guidelines on HREC membership, including member diversity and expertise.^2^ These changes support institutions to meet research governance responsibilities and remove the ambiguity surrounding HREC membership.^12^ Research may be eligible for exemption from ethics review if it is deemed to be of lower risk to participants and satisfies at least one of the following conditions^2^: (a) the research involves the use of collections of information or data from which all personal identifiers have been removed prior to being received by the researchers and where researchers explicitly agree; (b) the research is restricted to surveys and observation of public behaviour using information that was or will be collected and recorded without personal identifiers and is highly unlikely to cause distress to anyone associated with the information or the outcomes of the research; (c) is conducted as part of an educational training program in which the research activity is for training purposes only and where any outcomes or documentation are for program use only; and (d) the research uses only information that is publicly available through a mechanism set out by legislation or regulation and that is protected by law, such as mandatory reporting information, information obtained from registries of births and deaths, coronial investigations or reports of the Australian Bureau of Statistics. Some health promotion evaluations meeting these criteria may be eligible for exemption from ethical review, which may enable easier dissemination of evaluation results and other project findings. Practitioners should, however, be aware that organisations will need to grant an exemption for review. Institutions should clearly articulate policies and procedures for ethics review and approval as well as institutional authorisation of research. Journals may also request evidence of this exemption, a consideration for the HPJA moving forward. Positively, this change may facilitate bridging the previously identified gap in access to publications reporting on intervention effectiveness,^5^ which will build the evidence base for health promotion and better support evidence‐informed decision‐making. Regardless of exemption status, we encourage practitioners and organisations to familiarise themselves with the content of the National Statement and the ethical health promotion principles and values to guide their practice.^5^, ^7^, ^11^

Encouragingly, the updates to section 5 of the National Statement may support AHPA's endeavours to broker access to an ethical oversight mechanism sensitive to the health promotion context to address the aforementioned barriers to ethical health promotion practice in Australia.^5^ Changes may enable AHPA to establish its own formal ethics review process for lower‐risk health promotion projects and research, reviewed and supported by health promotion practitioners and researchers with a good understanding of the health promotion context. This may address previously identified barriers to ethical health promotion practice, including practitioners' challenges accessing formal oversight processes, and lack of context‐specific and sensitive HREC assessment.^5^

We anticipate further revisions to the National Statement in 2024, with potential additional implications for health promotion practice. The NHMRC is currently consulting on section 4: Ethical Considerations Specific to Participants, which covers population groups such as women who are pregnant, children and young people, people in dependent or unequal relationships, Aboriginal and Torres Strait Islander Peoples, and people in other countries.^2^ As much of health promotion practice involves working with diverse cultures and population groups, updates to this section of the National Statement will be of particular interest to the health promotion community, especially in light of the previously identified barrier of limited frameworks and guidelines to support meaningful engagement with priority populations.^5^ Updates to this section of the National Statement will enable sufficient guidance for the intended audiences (including researchers and HRECs) to address ethical considerations specific to these groups appropriately. Considering the perceived strong biomedical focus of many ethics guidelines and review processes found in previous research,^13^ the health promotion community will welcome updates to the National Statement that facilitate a more nuanced understanding of the complexities of working with different cultural and population groups outside a clinical setting.

We have highlighted key changes to the National Statement relevant to health promotion practice in Australia, considering previously identified barriers to ethical health promotion practice. There are a range of implications for individuals, health promotion agencies, organisations such as AHPA and academic journals such as the HPJA resulting from the changes. For example, AHPA will continue to build its capacity to lead conversations about ethical health promotion practices in Australia, including via our role as members of the HPEP. To support ethical practice, health promotion practitioners require an understanding and awareness of ethical frameworks and considerations for practice to assist with evidence‐informed decision‐making. We envision that familiarisation with the updated National Statement can support this process.

## CONFLICT OF INTEREST STATEMENT

KB is a Board member and GC is the National Vice‐President and Immediate Past President of AHPA. All authors are members of the HPEP Working Group and AHPA. KB is an Associate Editor of the HPJA. SB is Chair of the Curtin University Human Research Ethics Committee. Krysten Blackford is an Editorial Board member of HPJA and a co‐author of this article. To minimise bias, they were excluded from all editorial decision‐making related to the acceptance of this article for publication.

## Data Availability

Data sharing is not applicable to this article as no new data were created or analyzed in this study.
